# Supplementary material for: The terminal Ediacaran Tongshan Lagerstätte from South China
Source: Nat Commun. 2025 Nov 19;16:10161. doi: 10.1038/s41467-025-65176-2 (PMC12630636; doi:10.1038/s41467-025-65176-2)
Supplement: Supplementary file 1 — Supplementary Information [file 41467_2025_65176_MOESM1_ESM.pdf]

# Supplementary information

**The terminal Ediacaran Tongshan Lagerstätte from South China**

Jin-bo Hou[1]*, Xiang-dong Wang[1], Zhang-shuai Hou[1], Jahandar Ramezani[2], Qing Tang[2], Shu-zhong Shen[1]*

[1]State Key Laboratory of Critical Earth Material Recycling and Mineral Deposits, Frontiers Science Center for Critical Earth Material Cycling, School of Earth Sciences and Engineering, Nanjing University, Nanjing 210023, China.

[2]Department of Earth, Atmospheric and Planetary Sciences, Massachusetts Institute of Technology, Cambridge, MA 02139, USA

*Corresponding authors

## Supplementary Note 1: Social network analysis

Social network analysis without BST fronds shows that the Tongshan Lagerstätte shares more elements with Miaohe and Wenghui Lagerstätten (Supplementary Fig. 6d), which is largely affected by the habitats rather than by the evolutionary process because the Lagerstätten with the carbonaceous preservation have shared more elements with each other when compared to the Lagerstätten with the Ediacara-type preservation. Macroalgae are documented in a wide range of marine environments[1, 2]. Deep settings with low concentration of oxygen are, however, more favoring the preservation of macroalgae, such as a high diversity in the Lantian, Miaohe (or Wenghui), and Tongshan Lagerstätten, and the shallow settings with high concentration of oxygen are more favoring the preservation of metazoans such as the Ediacara, Shibantan and Gaojiashan Lagerstätten. This phenomenon is comparable to those Cambrian macroalgae that are least disturbed by metazoans in deep settings[3].

Higher metabolic rates are common in algae with simple growth forms, or high surface area to volume ratios, rather than systematic properties[4, 5]. The dominated filamentous algae (Fig. 2b–d, f, and i–k) and blade-like algae (Fig. 2a–e) in Tongshan Lagerstätte are characteristic of the algal morphologies with higher metabolic rates[4, 5] and represent a good indicator of a great amount of dissolved organic matter source, such as oxygen, particular organic carbon, dissolved organic carbon, and particular nitrogen, released for the local community. In such environments, metazoans (Figs. 3–6, Supplementary Fig. 6a–c) with lower oxygen demand could be likely satisfied.

## Supplementary Note 2: Geochemical and sedimentological evidence for the palaeoenvironment of the Tongshan Lagerstätte

EDS analyses of soft-bodied fronds (Table 1, Supplementary Fig. 7a–w) display high concentrations of C, Fe, P, and possibly S, which are contrasting to the high concentrations of Mg, Al, K, Ti, and Ba in the matrix. Si and O have similar concentrations in both body fossils and the matrix. Aluminosilicates could partially fill the voids left by soft tissues in BST fossils[6-10]. This explains why the body fossils have lower contents of aluminosilicate minerals than the matrix and indicates that the high concentrations of C, Fe, P, and possibly S, are true signals of body fossils. The presence of Fe in the body fossil is a direct indicator for exceptional preservation of soft-bodied fossils because irons react with sulfates to form pyrites, which can quickly replace the soft tissues at the early stage of decay[9, 11-13], allowing the preservation of soft tissues[14]. During subsequent weathering, original pyrites could form iron oxides and, at the same time, the sulfuric acids released due to this alteration were washed away. This explains the lower content of S detected in the body fossils and the reddish color of the fossils. The presence of S further indicates the microenvironments around body fossils during burial were partially anoxic, allowing iron in the water columns to react with the sulfates surrounding the decay materials. The

concentration of pyrites varies among Lagersätten. In the two most well-known BST Lagersätten, specimens of the middle Cambrian Burgess Shale Biota contain a lower proportion of pyrite compared to those of the lower Cambrian Chengjiang Biota [15]. The low proportion of pyrite in fonds makes the Tongshan Biota most comparable to the preservation condition of Burgess Shale Biota. In addition, the Tongshan Lagerstätte is preserved in siliceous shales/mudstones dominated by silicate minerals, which distinguishes it from most BST biotas preserved by early carbonate cements[16]. This condition is likely related to the widespread weathering of continental crust near the terminal Ediacaran[17-19].

The dark-coloured, thick clay-rich layers represent slow sedimentation of clay particles from the water column, corresponding to the background beds, while the light-coloured, thin silt-rich layers represent the event beds (Supplementary Fig. 8a–c). Cyclic changes between clay-rich and silt-rich beds reflect periodic fluctuations in local environmental energy, resulting in the rapid burial of organisms. These parallel beds without cross-stratification indicate that the deposition occurred below the storm wave base. Many well-preserved BST Lagerstätten such as Qingjiang[20] show similar depositional phenomena.

**Supplementary Note 3: Lateral continuity among the Ediacaran Lagerstätten**

Spread and diversification of organisms across the Ediacaran oceans at a given period are more complex compared to the knowing composition of communities and needs to consider the paleoenvironments, or facies, the organisms have occupied[21]. Both the Tongshan and Shibantan Lagerstätten show different compositions of organisms (Supplementary Fig. 6d, Supplementary Data 2) largely because of their paleoenvironmental difference, the former in deep carbonaceous muddy seafloor (below the storm wave base) located within the zone of low concentration of oxygen and the latter in shallow carbonate deposits [between fair-weather and storm wave bases[22]] located within the zone of high concentration of oxygen fueled possibly by microbial mats[23]. Each setting is selectively against particular organisms[24], indicating the taphonomic artefact has played an important role in understanding early organisms[21, 25].

Three assemblages of the Ediacara Biota shallowed up from the deep basin or slope (Avalon Assemblage) to the offshore settings (White Sea Assemblage), and then to the shoreface settings (Nama Assemblage) with time elapsed[25-27]. As paleoenvironments play a crucial role in the distribution of organisms[21, 24, 28], lacking the Lagerstätte representing the adjacent setting of each Ediacara Assemblage becomes difficult to make the distinguishment of pattern resulted from the biological turnover[27]. Deep settings such as Miaohe and Wenghui biotas are time-equivalent to Avalon and White Sea Assemblages (Fig. 1c). The deep setting Tongshan Lagerstätte reported herein is corresponding to the shallow-water setting Nama Assemblage (including the Shibantan Lagerstätte; Fig. 1c), providing a good reservoir of complementing

the missing biodiversity of the latest Ediacaran organisms lived in deep marine environments. The Tongshan and Shibantan Lagerstätten locate near to each other, about 350 km across, represent a laterally continuous community spreading from the shallow to deep settings, forming a unique window to look into the diversification of early organisms across the marine depth change happened in the terminal Ediacaran (Supplementary Fig. 9).

There is a possible transition between the Wenghui and Tongshan Lagerstätten. Wenghui Lagerstätte from the Doushantuo black shales was likely located in the transition zone between the open platform and the slope face[29, 30]. With the marine transgression happened in the late Ediacaran, the place where Wenghui Lagerstätte (>551 Ma) was located may become a deep siliceous basin[31] with time elapsed and simultaneously the Tongshan Lagerstätte (<551 Ma) succeed and became the one in the transitional zone between the platform and the siliceous basin[32]. At the same time, syndepositional extensional faulting along the south margin of the middle Yangtze in late Ediacaran could have released extensive hydrothermal silica into the seawater and deposited siliceous rocks[32].

**Supplementary Note 4: BST preservation in the Ediacaran period**

With respect to the Ediacara-type Lagerstätten that dominate the Ediacaran Era with a worldwide distribution[33], the Burgess Shale-type (BST) Lagerstätten are relatively rare in the Ediacaran[24, 34-42] even if they are quite common in the Cambrian or later periods[15, 43, 44]. The Ediacaran BST Lagerstätten are currently known from Brazil[45], South China[34, 37, 46], Western Mongolia[40], Namibia[47], India[41], Siberia[35, 36, 42], Urals[35], and United States[39].

BST preservation is relatively more common than the Ediacara-type preservation in early fossil Lagerstätten, of which the former can also yield more exquisite structural details[48, 49] when compared to the later[50]. However, in the Ediacaran Era the Ediacara-type preservation (casts and moulds) is largely favorable for the metazoans[33] and the BST (including carbonaceous) preservation is popular among macroalgae[34]. Understanding the Ediacaran organisms has been largely restricted by the preservational modes as the Ediacaran metazoans, predominantly knowing from the Ediacara-type preservation, are barely comparable to those Cambrian forms, predominantly knowing from the BST preservation. Diverse preservational modes have contributed to the exquisite preservation of early organisms in the Ediacaran period, such as Ediacara-type casts and moulds in siliciclastic rocks (Ediacara Biota) and limestones (Shibantan Biota), BST carbonaceous compressions in black shales (Lantian, Miaohe, Wenhui, Jiangchuan, and Wulingshan Biotas), and pyritizations in shales (Gaojiashan Biota). The distribution of different types of preservation for each biota intensifies the limitation of our ability to reveal the full morphology of organisms as each type only preserves the components it favors. Combination of two or more preservational styles can reveal more complete information of structures[51] but this opportunity is still very rare. For

example, the BST preservation can compensate the structural details that are lost in the Ediacara-type preservation as the cases of *Eoandromeda*, *Gesinella*, and so on[1, 52, 53]. In the Tongshan Lagerstätte all the preservational styles mentioned above, the BST preservation (including carbonaceous compression) (Figs. 2, 3), the pyritization (Figs. 4–6, Supplementary Fig. 4c), and the possible Ediacara-type preservation (Supplementary Fig. 5a–d), are occurred. Two or more preservational modes are even present in the same species such as *B. taniata* with three modes (including carbonaceous compression) (Supplementary Fig. 4a–c), *B. brunsae* with two modes (Fig. 2h, Supplementary Fig. 5a–d), and *P. linearis* with two modes (Fig. 3c, Supplementary Fig. 6a). Integrating diverse preservational evidence available in this Lagerstätte would provide more complete information of organisms in understanding the early evolution of life.

## Supplementary Figures

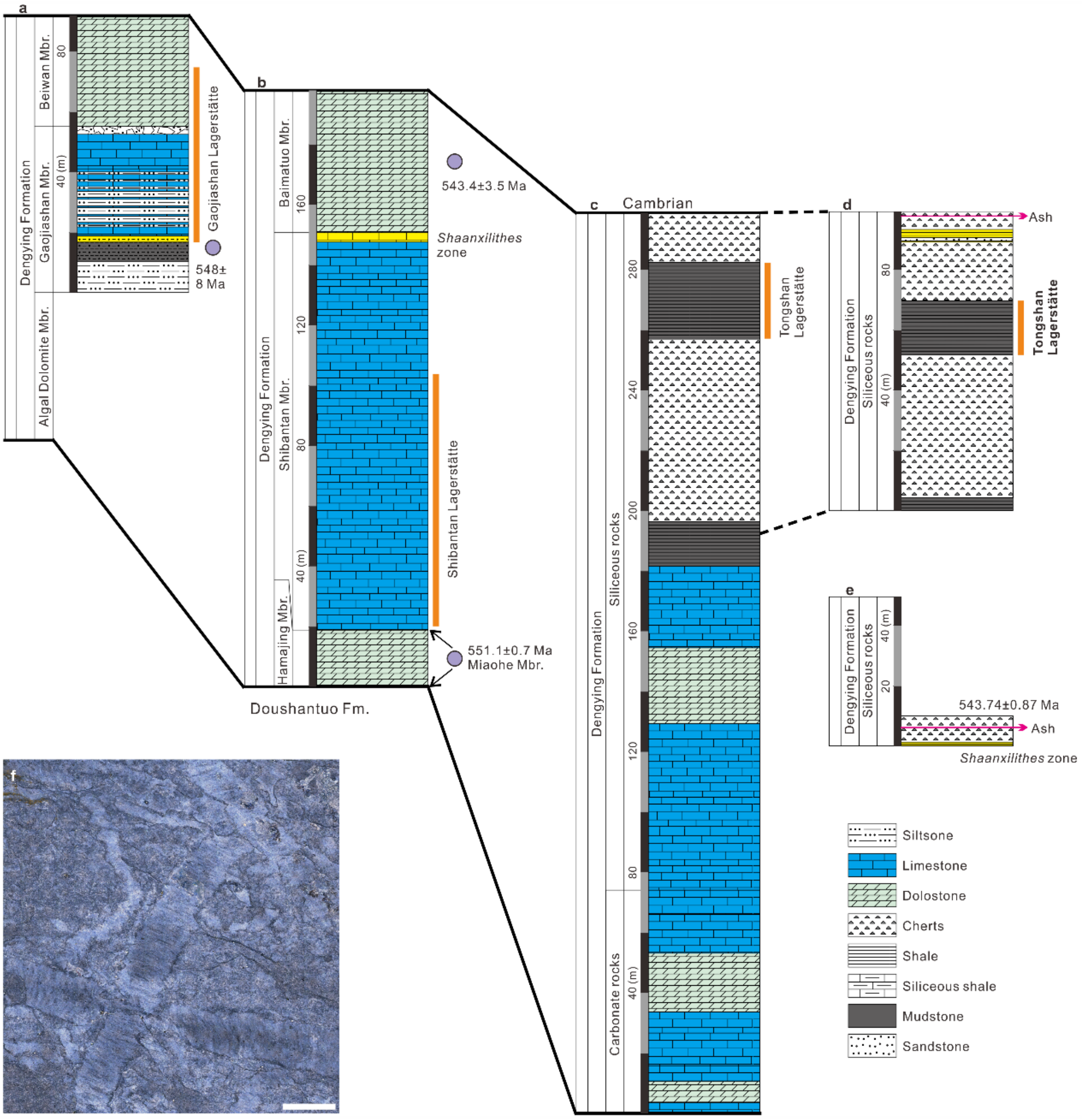


**Supplementary Figure 1 | Stratigraphic correlation of Gaojiashan, Shibantan, and Tongshan Lagerstätten. a** Gaojiashan section modified from Cai et al.[54] (permitted by Y. Cai) and zircon dating collected from Cui et al.[55]. **b** Zhelinziwan section modified from An et al.[56] (permitted by Z. An) and integrated with information from Xiao et al.[57]. **c** Composite stratigraphy of the Dengying Formation in the Hefeng-Tongshan area, Hubei Province, South China[58]; lower limestones were measured at Shimentang Reservoir of the Tongshan County, and top siliceous rocks were measured at Xizhuang village of the Tongshan County[58]. **d** Wanjia section, Tongshan County, Hubei Province, South China; an ash layer marked with a pink arrow has zircons preserved with high quality and is likely the same ash layer discovered at the Jiweijian section. **e** Jiweijian section, Tongshan County, Hubei Province, South China; an ash layer marked with a pink arrow above the *Shaanxilithes* zone yields the zircons for age measurement in this study. **f** *Shaanxilithes ningqingensis* at the Jiweijian section, Tongshan County, South China; scale bar is 5 mm in length, ESEN

0022. *Shaanxilithes* zone is marked with yellow background.

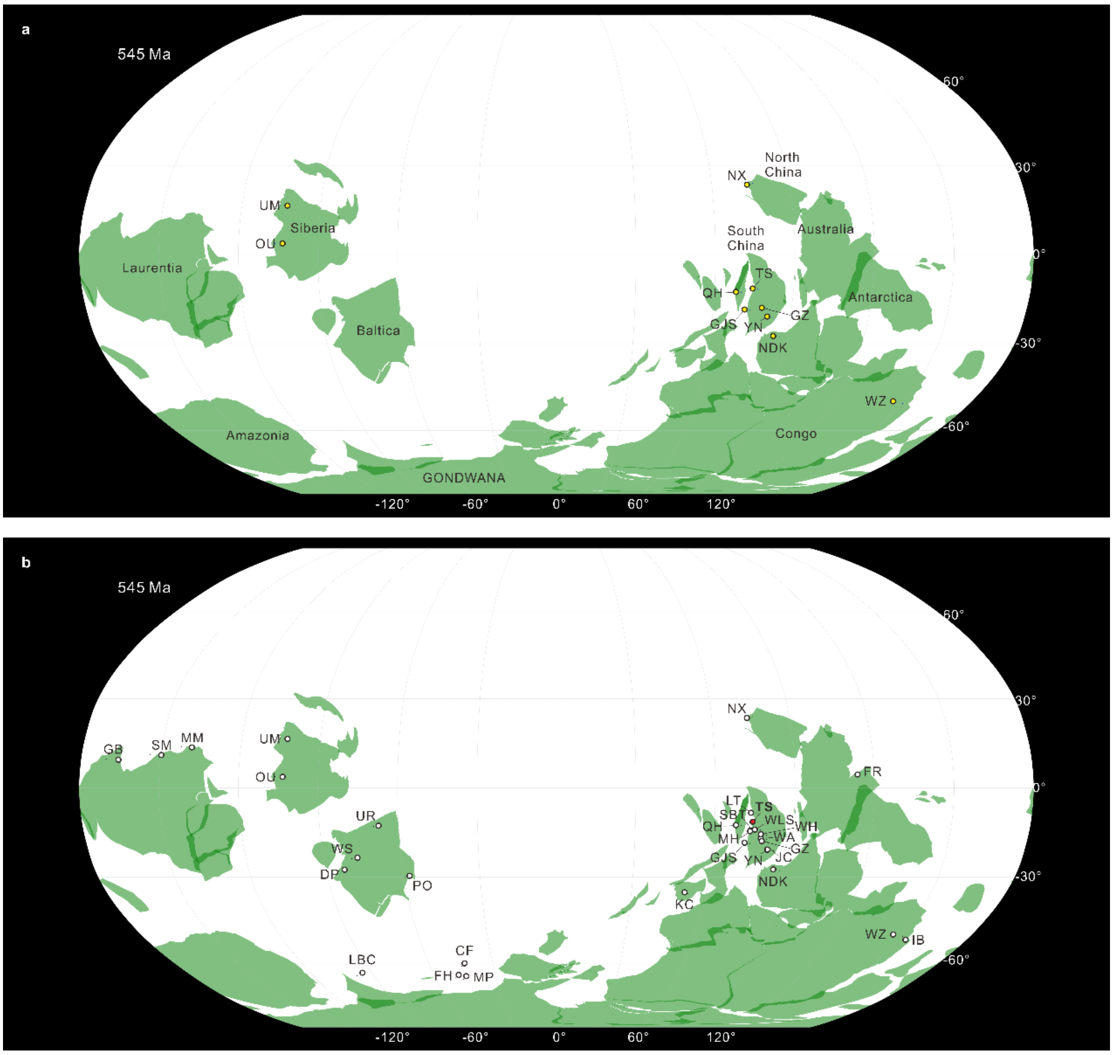


**Supplementary Figure 2 | Worldwide distribution of the potential index fossil *Shannixilithes* and the Ediacara-type fossils. a** Global distribution of *Shaanxilithes* showing on the continental reconstruction, created from GPlates software[59]. Localities of *Shaanxilithes* in China are represented by six provinces: Gouzhou (GZ), Hubei (HB), Ningxia (NX), Qinghai (QH), Shaanxi (SX), and Yunnan (YN). **b** Global distribution of typical Ediacara-type fossils[33] and other Ediacaran biotas discovered in China. Abbreviations of the Ediacaran biotas discovered in China: GJS, Gaojiashan biota: JC, Jiangchuan biota; LT, Liantian biota; MH, Miaohe biota; SBT, Shibantan biota; TS, Tongshan biota; WA, Weng'an biota; WH, Wenghui biota. Abbreviations of typical Ediacara-type fossil localities: CF, Charnwood Forest, UK; DP, Digermull Peninsula, Norway; FH, Fortune Head, Canada; FR, Flinders Ranges, Australia; GB, Great Basin, USA; IB, Itajai Basin, Brazil; KC, Kushk and Chahmir, Iran; LBC, Little Bear Creek, USA; MM, Mackenzie Mountains, Canada; NDK, Nigali Dhar Korgai, India; OU, Olenek Uplift, Russia; PO, Podolia, Ukraine; SM, Salient Mountains and Mount Fotzwilliam, Canada; WS, White Sea, Russia; WZ, Witputs

Subbasin and Zaris Subbasin, Namibia; UM, Uchur-Maya, Russia; UR, Urals, Russia.

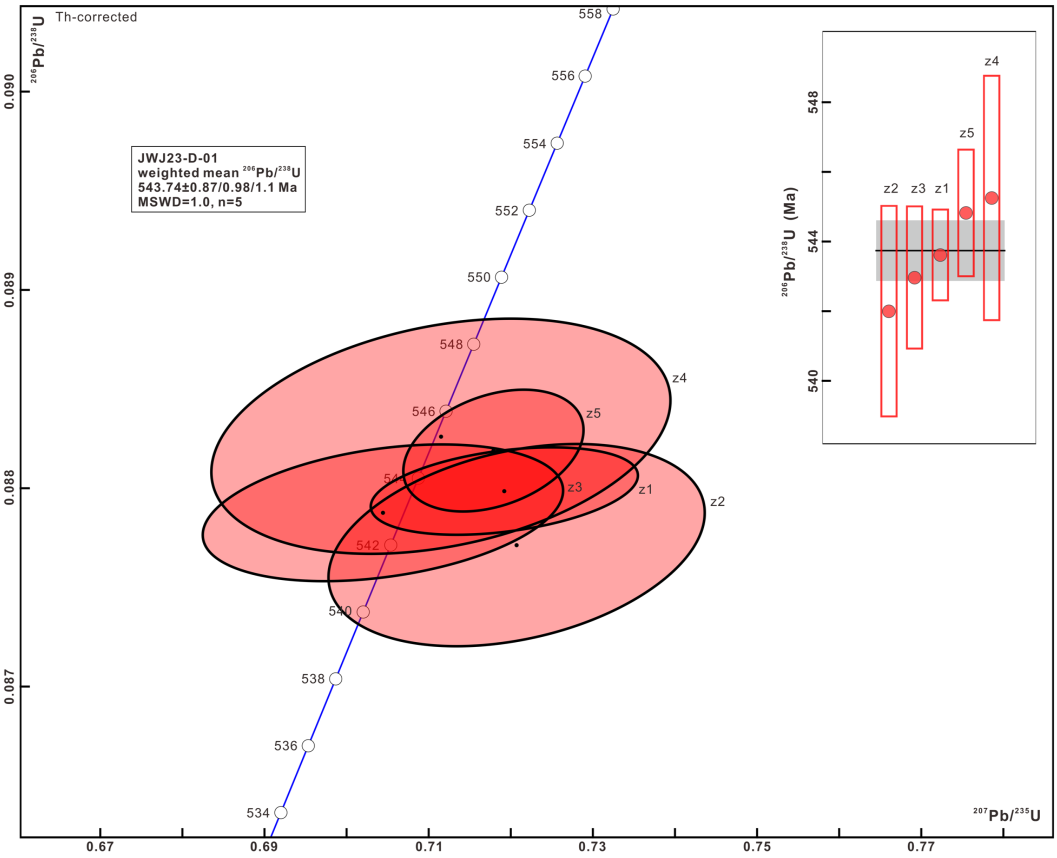


**Supplementary Figure 3 | U-Pb zircon geochronology from the Jiweijian section, Tongshan County, South China.** The conventional concordia plot for sample JWJ23-D-01 shows individual zircon analyses with their 2σ error ellipses, and all individual analyses (n=5 zircons) intersect the concordia line and are therefore concordant. The inset rank-order plot of the zircon $^{206}$Pb/$^{238}$U dates shows individual zircon age results with 2σ uncertainty marked with grey background, and the horizontal black line with shaded band signifies the calculated weighted mean $^{206}$Pb/$^{238}$U age with 2σ error (*X/Y/Z*) (p-value = 0.39). z1 to z5 in the figure represent five zircon crystals analyzed using the CA-ID-TIMS method, and each was analyzed once.

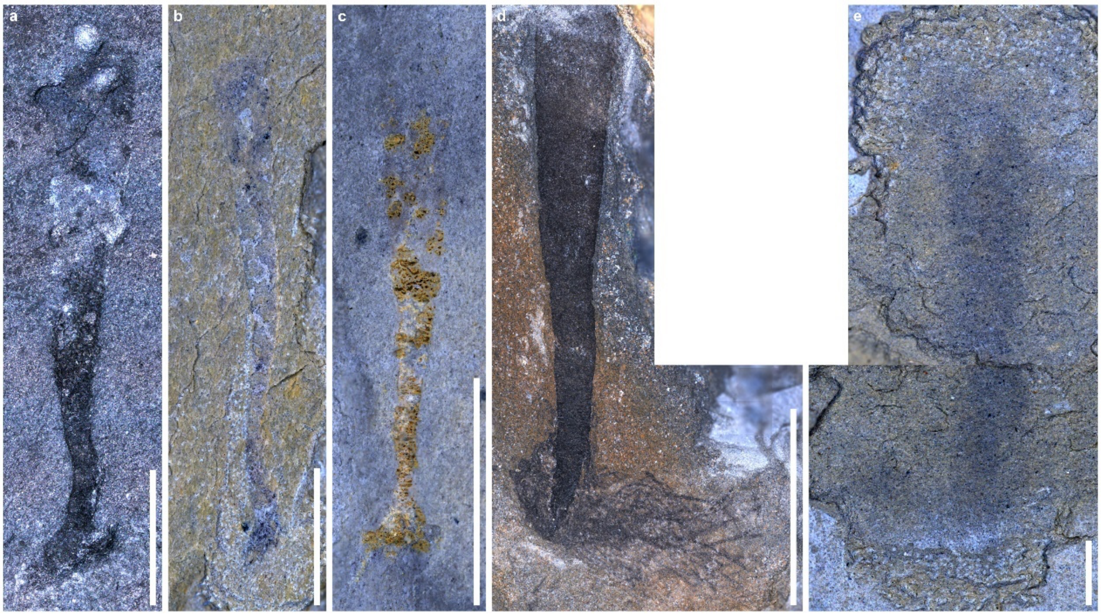


**Supplementary Figure 4 | Macroalgae with multiple preservational types.** **a** Burgess Shale-type preservation of *Baculiphyca taniata*, ESEN 0023. **b** carbonaceous *Baculiphyca taniata*, ESEN 0024. **c** Pyritized *Bauculiphyca taniata*, ESEN 0025. **d** Carbonaceous ?*Gesinella hunanensis*, ESEN 0026. **e** Carbonaceous U-shaped *Tawuia* which is artificially connected by two specimens, the part ESEN 0027 at the top and the counterpart ESEN 0028 with reversed image at the bottom. Scale bars in **a** and **e** = 2 mm, in **b** and **c** = 5 mm and in **d** = 10 mm.

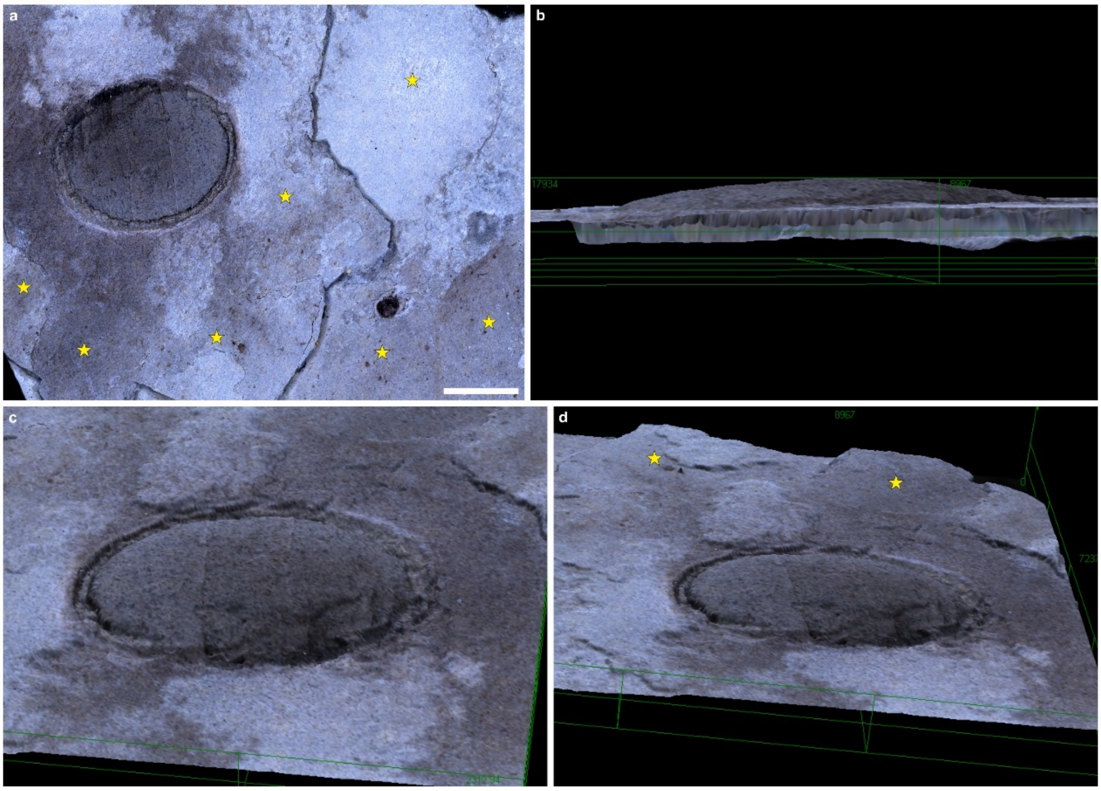

**Supplementary Figure 5 | Two preservational types present in *Beltanelliformis brunsae*. a** Ediacara-type *B. brunsae* and other carbonaceous individuals marked with yellow stars, ESEN 0029. **b** Lateral view of the Ediacara-type *B. brunsae*. **c** Tilted view to show the relief of the Ediacara-type *B. brunsae*. **d** Tilted view with a different angle to show the relief of the Ediacara-type *B. runsae*. Scale bar is 5 mm in length.

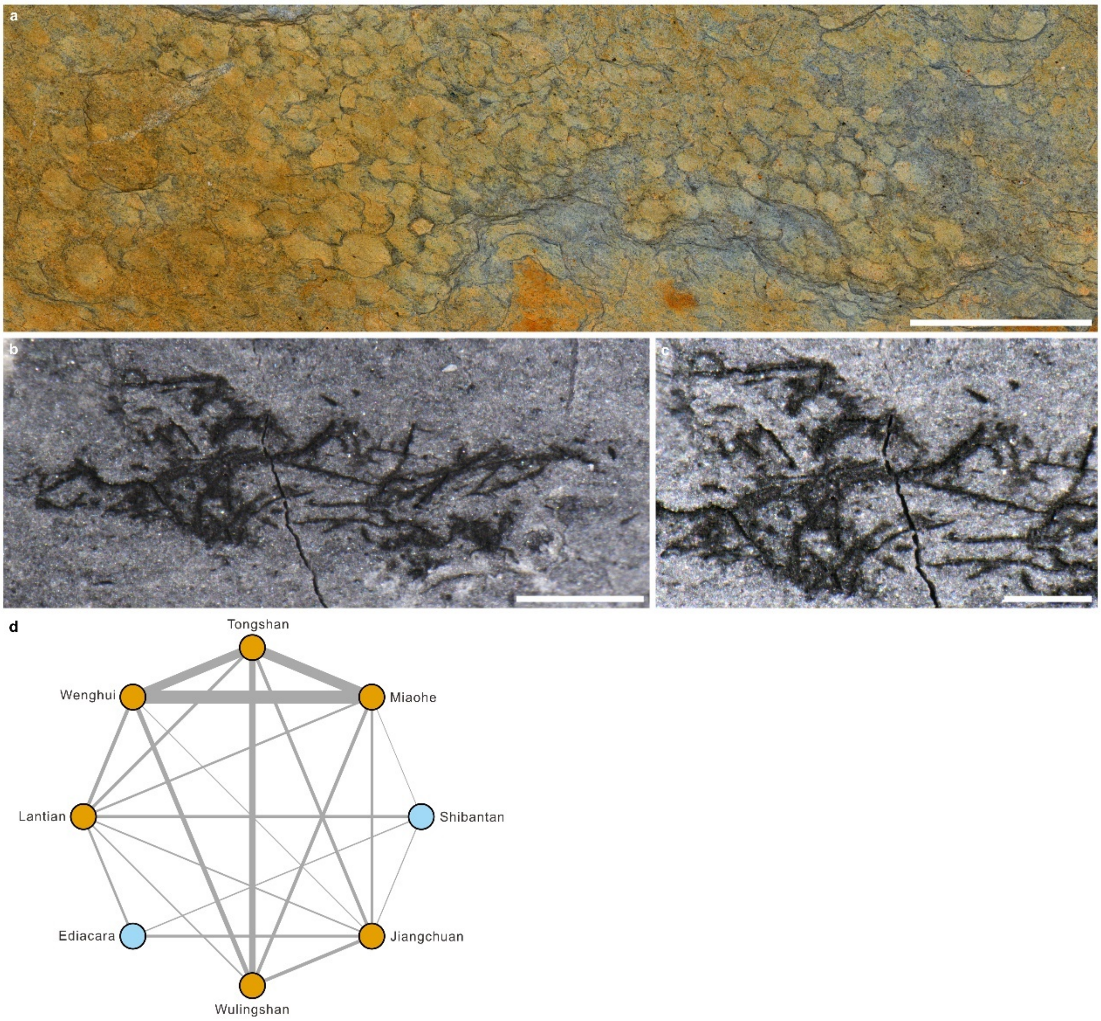


**Supplementary Figure 6 | *Palaeopascichnus* and *Sinospongia* in the Tongshan Lagerstätte and social network analysis of BST Ediacaran Lagerstätten. a** Pyritized *Palaeopascichnus linearis*, ESEN 0030. **b–c** *Sinospongia chenjunyuani*, ESEN 0031. **d** Social network analysis showing correlations of the BST Ediacaran Lagerstätten discovered in South China; The Ediacara Biota is applied to show that a rare number of species are shared among BST (marked with orange color) and Ediacara-type (marked with ice blue color) Lagerstätten. Scale bars in **a** = 10 mm, in **b** = 1 mm and in **c** = 0.5 mm.

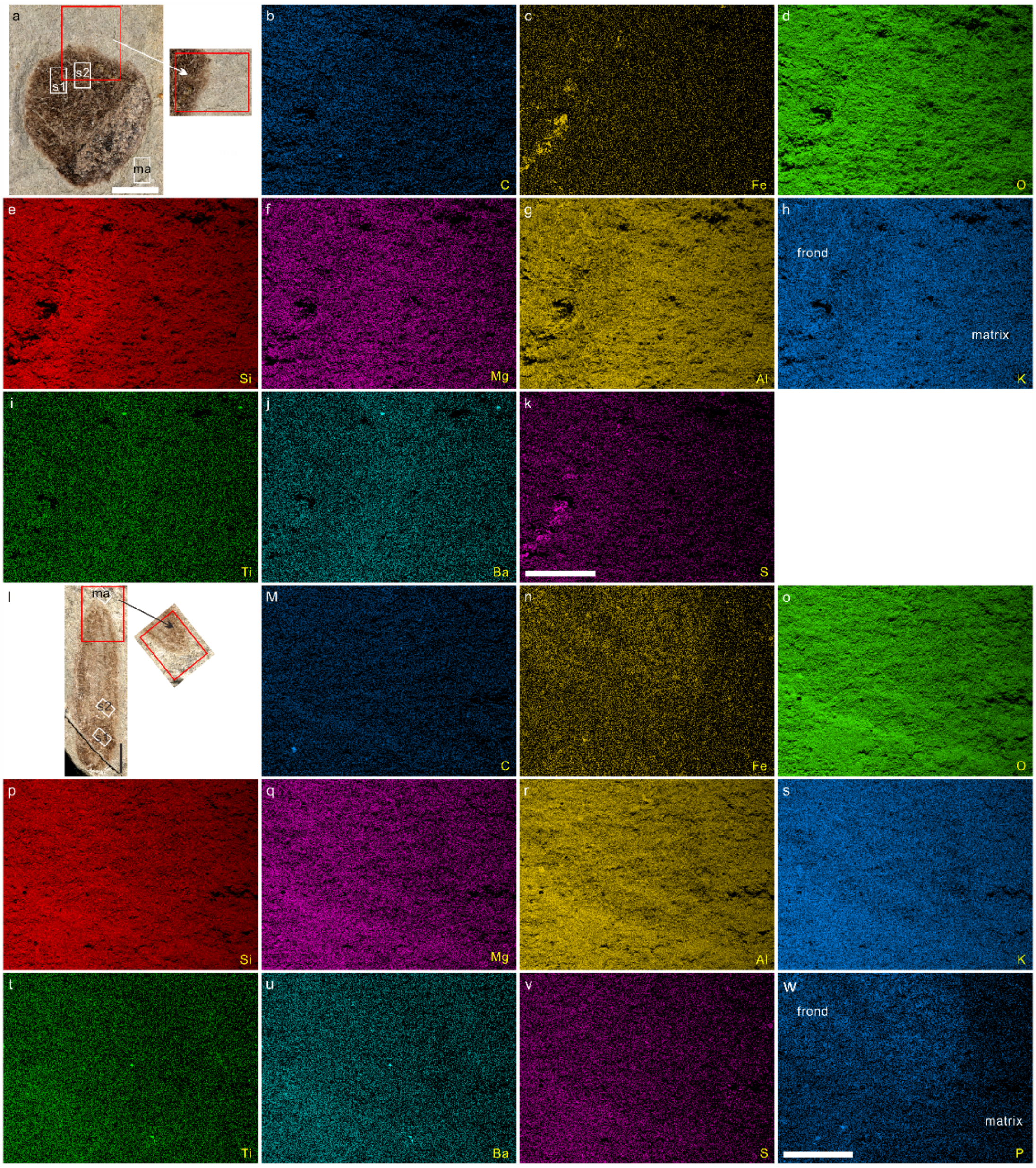


**Supplementary Figure 7 | Elemental mapping of BST fronds in the Tongshan Lagerstätte. a** ESEN 0017 showing four detected sites of EDS analysis. **b**–**k**, EDS elemental maps of the site outlined with a red box in figure a. **l** ESEN 0019a showing four detected sites of EDS analysis. **m–w** EDS elemental maps of the site outlined with a red box in figure l. Each site was mapped once. Abbreviations: s1–s2, detected sites 1 and 2 on the frond body, respectively; ma, detected site on the matrix. Scale bars in **a** and **l** = 2 mm and in **b**–**k** and **m**–**w** = 1 mm.

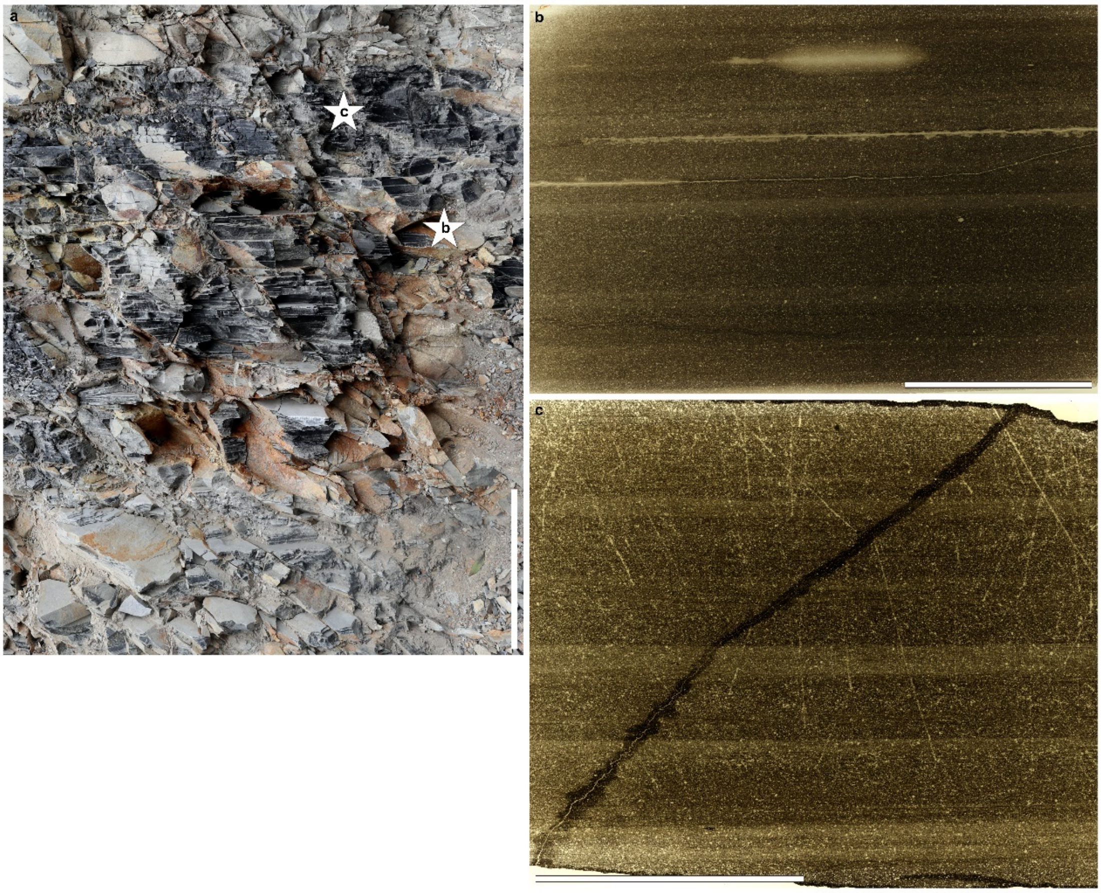


**Supplementary Figure 8 | Well laminated beds of the Tongshan Lagersattte. a** A field image showing laminated carbonaceous shales/mudstones, in which gray lines or surfaces represent weathered layers and two white stars mark the intervals from which two thin-sections were collected. **b** Thin-section photomicrograph from the interval in figure a, showing thick clay-rich layers interval with thin silt-rich layers. **c** Thin-section photomicrograph from the interval in figure a, showing thick clay-rich layers interval with thin silt-rich layers; many dark and discontinuous lines represent organismal remains. Scale bars in **a** = 20 cm and in **b** and **c** = 1 cm.

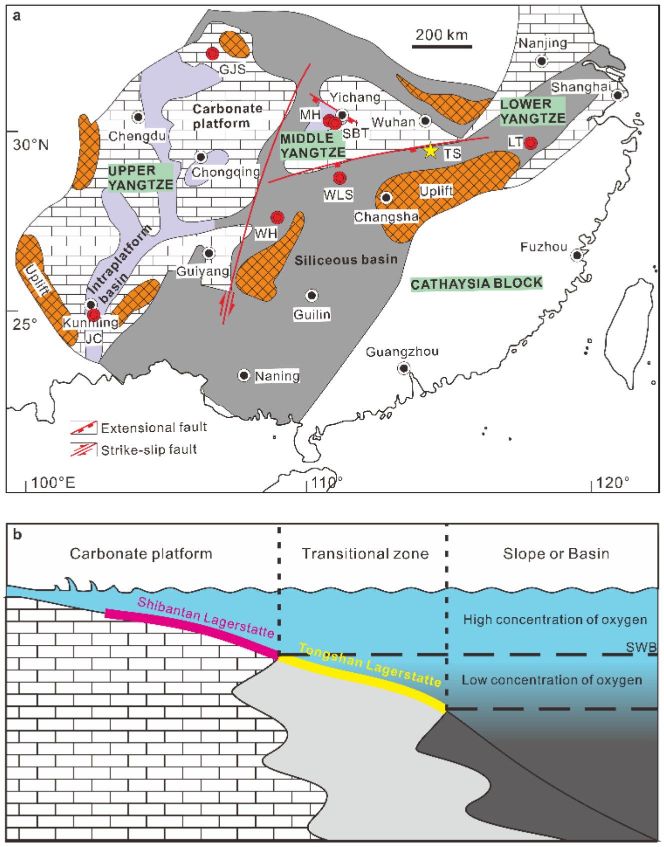


**Supplementary Figure 9 | Reconstruction of the palaeoenvironment of the Tongshan Lagerstätte. a** Tectono-depositional reconstruction of the Yangtze Block during latest Ediacaran period (551–542 Ma) (modified from[32] and permitted by D. Chen); The Tongshan Lagerstätte marked with a yellow star locates in the transition zone between the carbonate platform and the siliceous basin[60-62], or on the marginal siliceous basin[32]. **b** Reconstruction of the lateral continuity of the Tongshan and Shibantan Lagerstätten; Tongshan Lagerstätte locates in a relatively deep marine setting compared to the Shibantan Lagerstätte.

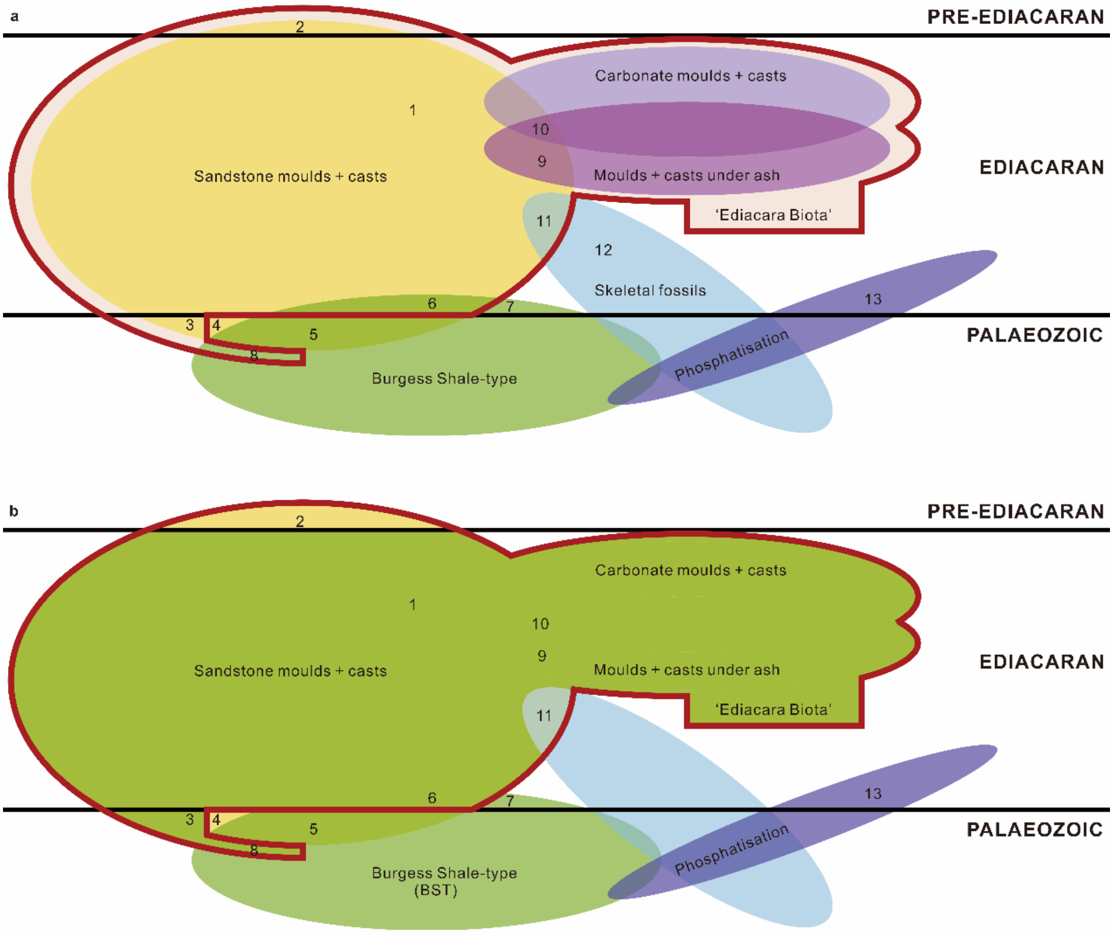


**Supplementary Figure 10 | Mouldic and Burgess Shale-type preservations of the Ediacara Biota. a** The traditional view of the Ediacara biota, outlined with a thick red line, is that it is predominately preserved as moulds and casts[63], which represent the only preservation style for frondose animals—the dominant members of Ediacara Biota; this modification is permitted by Geoscience Frontiers. **b** The current view of the Ediacara Biota in this study is based on typical Ediacaran fronds recovered from the Tongshan Lagerstätte, which expand the mouldic preservation of Ediacara Biota with the BST preservation. The current view shows that the mouldic and BST preservation styles represent the general conditions for the Ediacara Biota and Ediacaran fossils originally represented as number 6 in figure a. Numbers represent particular fossils or groups of fossils: 1, non-mineralized Ediacaran mouldic fossils; 2, pre-Ediacaran fossils preserved as moulds and casts; 3, Palaeozoic fronds with mouldic preservation; 4, other mouldic Palaeozoic fossils; 5, Palaeozoic fossils with both mouldic and BST preservations; 6, Ediacaran fossils with both mouldic and BST preservations; 7, Ediacaran fossils only with the BST preservation; 8, Palaeozoic fossils only with the BST preservation; 9, typical Ediacara fronds with mouldic preservation in ash beds; 10, typical Edaicara fronds with mouldic preservation in ash beds and carbonates; 11, Ediacaran skeletal fossils with mouldic preservation; 12, other Ediacaran skeletal fossils;

13, phosphatized fossils. This diagram and contents are modified from MacGabhann[63]. This modification is permitted by Geoscience Frontiers.

**Supplementary References**


1. Xiao S*, et al.* Affirming life aquatic for the Ediacara biota in China and Australia. *Geology* **41**, 1095–1098 (2013).

2. Xiao S, Gehling JG, Evans SD, Hughes IV, Droser ML. Probable benthic macroalgae from the Ediacara Member, South Australia. *Precambrian Res* **350**, 105903 (2020).

3. Lan T, Yang J, Zhang X-g, Hou J-b. A new macroalgal assemblage from the Xiaoshiba Biota (Cambrian Series 2, Stage 3) of southern China. *Palaeogeogr Palaeoclimatol Palaeoecol* **499**, 35–44 (2018).

4. Haas AF, Naumann MS, Struck U, Mayr C, el-Zibdah M, Wild C. Organic matter release by coral reef associated benthic algae in the Northern Red Sea. *J Exp Mar Biol Ecol* **389**, 53–60 (2010).

5. Haas AF*, et al.* Effects of coral reef benthic primary producers on dissolved organic carbon and microbial activity. *PLoS One* **6**, e27973 (2011).

6. Orr PJ, Briggs DEG, Kearns SL. Cambrian Burgess Shale animals replicated in clay minerals. *Science* **281**, 1173–1175 (1998).

7. Butterfield NJ, Balthasar U, Wilson LA. Fossil diagenesis in the Burgess Shale. *Palaeontology* **50**, 537–543 (2007).

8. Orr PJ, Kearns SL, Briggs DEG. Elemental mapping of exceptionally preserved 'carbonaceous compression' fossils. *Palaeogeogr Palaeoclimatol Palaeoecol* **277**, 1–8 (2009).

9. Gaines RR. Burgess Shale-type preservation and its distribution in space and time. *The Paleontological Society Papers* **20**, 123–146 (2014).

10. Gaines RR, Lombardo AJ, Holzer IO, Caron J-B. The limits of burgess shale-type preservation: assessing the evidence for preservation of the blood protein hemocyanin in the burgess shale. *Palaios* **34**, 291–299 (2019).

11. Briggs DEG, Bottrell SH, Raiswell R. Pyritization of soft-bodied fossils: Beecher's Trilobite Bed, Upper Ordovician, New York State. *Geology* **19**, 1221–1224 (1991).

12. Briggs DEG, Raiswell R, Bottrell SH, Hatfield D, Bartels C. Controls on the pyritization of exceptionally preserved fossils; an analysis of the

Lower Devonian Hunsrueck Slate of Germany. *Am J Sci* **296**, 633–663 (1996).

13. Gabbott SE, Xian-guang H, Norry MJ, Siveter DJ. Preservation of Early Cambrian animals of the Chengjiang biota. *Geology* **32**, 901 (2004).

14. Gibson BM, Schiffbauer JD, Wallace AF, Darroch SAF. The role of iron in the formation of Ediacaran 'death masks'. *Geobiology* **21**, 421–434 (2023).

15. Gaines RR, Briggs DEG, Zhao YL. Cambrian Burgess Shale–type deposits share a common mode of fossilization. *Geology* **36**, 755 (2008).

16. Gaines RR*, et al.* Mechanism for Burgess Shale-type preservation. *Proc Natl Acad Sci USA* **109**, 5180-5184 (2012).

17. Kennedy M, Droser M, Mayer LM, Pevear D, Mrofka D. Late Precambrian oxygenation; inception of the clay mineral factory. *Science* **311**, 1446–1449 (2006).

18. Peters SE, Gaines RR. Formation of the 'Great Unconformity' as a trigger for the Cambrian explosion. *Nature* **484**, 363-366 (2012).

19. Wei G-Y*, et al.* Lithium isotopic constraints on the evolution of continental clay mineral factory and marine oxygenation in the earliest Paleozoic Era. *Sci Adv* **10**, eadk2152 (2024).

20. Fu D*, et al.* The Qingjiang biota—a Burgess Shale–type fossil Lagerstätte from the early Cambrian of South China. *Science* **363**, 1338–1342 (2019).

21. Gehling JG, Droser ML. How well do fossil assemblages of the Ediacara Biota tell time? *Geology* **41**, 447–450 (2013).

22. Chen Z*, et al.* New Ediacara fossils preserved in marine limestone and their ecological implications. *Sci Rep* **4**, 4180 (2014).

23. Huang K*, et al.* Interaction of Shibantan Biota and environment in the terminal Ediacaran ocean: Evidence from I/(Ca+ Mg) and sulfur isotopes. *Precambrian Res* **379**, 106814 (2022).

24. Boag TH, Darroch SAF, Laflamme M. Ediacaran distributions in space and time: testing assemblage concepts of earliest macroscopic body fossils. *Paleobiology* **42**, 574–594 (2016).

25. Bowyer FT, Wood RA, Yilales M. Sea level controls on Ediacaran-Cambrian animal radiations. *Sci Adv* **10**, eado6462 (2024).

26. Droser ML, Tarhan LG, Gehling JG. The rise of animals in a changing environment: global ecological innovation in the late Ediacaran. *Annu Rev Earth Planet Sci* **45**, 593–617 (2017).

27. Muscente A, Boag TH, Bykova N, Schiffbauer JD. Environmental disturbance, resource availability, and biologic turnover at the dawn of animal life. *Earth Sci Rev* **177**, 248–264 (2018).

28. Droser ML*, et al.* What happens between depositional events, stays between depositional Events: the Significance of organic mat surfaces in the capture of Ediacara communities and the sedimentary rocks that preserve them. *Front Earth Sci* **10**, 826353 (2022).

29. Wang Y*, et al.* Preliminary discussion on ecological characteristics and buried environments of Miaohe-type Biota during the Late Doushantuoian of Sinian in northeastern Guizhou Province. *J Palaeogeogr* **7**, 327–335 (2005).

30. Jiang G, Shi X, Zhang S, Wang Y, Xiao S. Stratigraphy and paleogeography of the Ediacaran Doushantuo Formation (ca. 635–551Ma) in South China. *Gondwana Res* **19**, 831-849 (2011).

31. Deng L*, et al.* Environmental drivers of biotic turnover: Insight from tectono-sedimentary environment transition during the terminal Ediacaran to Early Cambrian. *Precambrian Res* **417**, 107666 (2025).

32. Ding Y, Chen D, Zhou X, Guo C, Huang T, Zhang G. Tectono-depositional pattern and evolution of the middle Yangtze Platform (South China) during the late Ediacaran. *Precambrian Res* **333**, 105426 (2019).

33. Evans SD*, et al.* Environmental drivers of the first major animal extinction across the Ediacaran White Sea-Nama transition. *Proc Natl Acad Sci USA* **119**, e2207475119 (2022).

34. Xiao SH, Yuan XL, Steiner M, Knoll AH. Macroscopic carbonaceous compressions in a terminal Proterozoic shale: A systematic reassessment of the Miaohe Biota, South China. *J Paleontol* **76**, 347–376 (2002).

35. Grazhdankin DV, Krayushkin AV. Trace fossils and the Upper Vendian

boundary in the southeastern White Sea region. *Dokl Earth Sci* **416**, 1027–1031 (2007).

36. Grazhdankin DV, Balthasar U, Nagovitsin KE, Kochnev BB. Carbonate-hosted Avalon-type fossils in arctic Siberia. *Geology* **36**, 803–806 (2008).

37. Yuan X, Chen Z, Xiao S, Zhou C, Hua H. An early Ediacaran assemblage of macroscopic and morphologically differentiated eukaryotes. *Nature* **470**, 390–393 (2011).

38. Tarhan LG*, et al.* Precambrian-Cambrian boundary interval occurrence and form of the enigmatic tubular body fossil *Shaanxilithes ningqiangensis* from the Lesser Himalaya of India. *Palaeontology* **57**, 283–298 (2014).

39. Rowland SM, Rodriguez MG. A multicellular alga with exceptional preservation from the Ediacaran of Nevada. *J Paleontol* **88**, 263–268 (2014).

40. Dornbos SQ, Oji T, Kanayama A, Gonchigdorj S. A new Burgess Shale-type deposit from the Ediacaran of western Mongolia. *Sci Rep* **6**, 23438 (2016).

41. Sharma M*, et al.* Palaeobiology of Indian Proterozoic and early Cambrian successions—recent developments. *Proc Indian Natl Sci Acad* **82**, 559–579 (2016).

42. Petrov PY, Vorob'eva NG. Representatives of the Miaohe Biota from the Ediacaran (Vendian) Pre-Shuram Strata of the Patom Highland, Siberia. *Stratigr Geol Correl* **30**, 52–64 (2022).

43. Conway Morris S. Burgess Shale faunas and the Cambrian explosion. *Science* **246**, 339–346 (1989).

44. Anderson RP, Woltz CR, Tosca NJ, Porter SM, Briggs DE. Fossilisation processes and our reading of animal antiquity. *Trends Ecol Evol* **38**, 1060-1071 (2023).

45. Becker-Kerber B*, et al.* Clay templates in Ediacaran vendotaeniaceans: Implications for the taphonomy of carbonaceous fossils. *Geol Soc Am Bull* **134**, 1334–1346 (2022).

46. Cai Y, Schiffbauer JD, Hua H, Xiao S. Preservational modes in the Ediacaran Gaojiashan Lagerstätte: Pyritization, aluminosilicification, and

carbonaceous compression. *Palaeogeogr Palaeoclimatol Palaeoecol* **326-328**, 109-117 (2012).

47. Leonov MV, Fedonkin MA, Vichers-Rich P, Ivantsov AY, Trusler P. Discovery of the first macroscopic algal assemblage in the terminal Proterozoic of Namibia, southwest Africa. *Communications of the Geological Survey of Namibia* **14**, 87–93 (2009).

48. Briggs DEG, Erwin DH, Collier FJ. *The Fossils of the Burgess Shale*. Smithsonian Institution Press (1994).

49. Gaines RR*, et al.* Burgess shale-type biotas were not entirely burrowed away. *Geology* **40**, 283-286 (2012).

50. MacGabhann BA*, et al.* Resolution of the earliest metazoan record: Differential taphonomy of Ediacaran and Paleozoic fossil molds and casts. *Palaeogeogr Palaeoclimatol Palaeoecol* **513**, 146–165 (2019).

51. Kenchington CG, Wilby PR. Of time and taphonomy: preservation in the Ediacaran. *The Paleontological Society Papers* **20**, 101–122 (2014).

52. Zhu M, Gehling JG, Xiao S, Zhao Y, Droser ML. Eight-armed Ediacara fossil preserved in contrasting taphonomic windows from China and Australia. *Geology* **36**, 867–870 (2008).

53. Wan B*, et al.* A tale of three taphonomic modes: the Ediacaran fossil *Flabellophyton* preserved in limestone, black shale, and sandstone. *Gondwana Res* **84**, 296–314 (2020).

54. Cai Y, Hua H, Xiao S, Schiffbauer JD, Li P. Biostratinomy of the late Ediacaran pyritized Gaojiashan Lagerstätte from southern Shaanxi, south China: importance of event deposits. *Palaios* **25**, 487–506 (2010).

55. Cui H*, et al.* Environmental context for the terminal Ediacaran biomineralization of animals. *Geobiology* **14**, 344–363 (2016).

56. An Z-h, Zhao X-m, Niu Z-j, Li Z-h, Ye Q. Discovery of Shaanxilithes from the Dengying Formation in the Yangtze Gorges area, South China, and its stratigraphic significance. *China Geol* **4**, 649–651 (2020).

57. Xiao S, Chen Z, Pang K, Zhou C, Yuan X. The Shibantan Lagerstätte: insights into the Proterozoic–Phanerozoic transition. *J Geol Soc* **178**, jgs2020-2135 (2020).

58. Province BBoGaMRoH. *Regional Geology of Hubei Province*. Geological Publishing House (1990).

59. Merdith AS*, et al.* Extending full-plate tectonic models into deep time: Linking the Neoproterozoic and the Phanerozoic. *Earth Sci Rev* **214**, 103477 (2021).

60. Zhu M, Zhang J, Yang A, Li G, Steiner M, Erdtmann BD. Sinian-Cambrian stratigraphic framework for shallow-to deep-water environments of the Yangtze Platform: an integrated approach. *Prog Nat Sci* **13**, 951–960 (2003).

61. Chen D, Wang J, Qing H, Yan D, Li R. Hydrothermal venting activities in the Early Cambrian, South China: Petrological, geochronological and stable isotopic constraints. *Chem Geol* **258**, 168–181 (2009).

62. Yang C, Zhu M, Condon DJ, Li X-H. Geochronological constraints on stratigraphic correlation and oceanic oxygenation in Ediacaran-Cambrian transition in South China. *J Asian Earth Sci* **140**, 75–81 (2017).

63. MacGabhann BA. There is no such thing as the ‘Ediacara Biota’. *Geoscience Frontiers* **5**, 53–62 (2014).
