# Supplementary material for: The terminal Ediacaran Tongshan Lagerstätte from South China
Source: Nat Commun. 2025 Nov 19;16:10161. doi: 10.1038/s41467-025-65176-2 (PMC12630636; doi:10.1038/s41467-025-65176-2)
Supplement: Supplementary file 2 — Description of Addtional Supplementary Files [file 41467_2025_65176_MOESM2_ESM.pdf]

Description of Additional Supplementary Files

File Name: Supplementary Data 1
Description: U-Pb isotopic data.

File Name: Supplementary Data 2
Description: Dataset for social network analysis. Macro-organisms documented in the Lantian, Miaohe, Wenghui, Shibantan, Jiangchuan, Wulingshan, and Tongshan Lagerstätten.
