# Supplementary material for: The terminal Ediacaran Tongshan Lagerstätte from South China
Source: Nat Commun. 2025 Nov 19;16:10161. doi: 10.1038/s41467-025-65176-2 (PMC12630636; doi:10.1038/s41467-025-65176-2)
Supplement: Supplementary file 5 — Reporting Summary [file 41467_2025_65176_MOESM5_ESM.pdf]

nature portfolio

Corresponding author(s): Jin-bo Hou, Shu-zhong Shen

Last updated by author(s): Sep 26, 2025

Reporting Summary

Nature Portfolio wishes to improve the reproducibility of the work that we publish. This form provides structure for consistency and transparency in reporting. For further information on Nature Portfolio policies, see our Editorial Policies and the Editorial Policy Checklist.

Statistics

For all statistical analyses, confirm that the following items are present in the figure legend, table legend, main text, or Methods section.

n/a	Confirmed	
☐	☒	The exact sample size (*n*) for each experimental group/condition, given as a discrete number and unit of measurement
☒	☐	A statement on whether measurements were taken from distinct samples or whether the same sample was measured repeatedly
☒	☐	The statistical test(s) used AND whether they are one- or two-sided<br>*Only common tests should be described solely by name; describe more complex techniques in the Methods section.*
☒	☐	A description of all covariates tested
☒	☐	A description of any assumptions or corrections, such as tests of normality and adjustment for multiple comparisons
☒	☐	A full description of the statistical parameters including central tendency (e.g. means) or other basic estimates (e.g. regression coefficient) AND variation (e.g. standard deviation) or associated estimates of uncertainty (e.g. confidence intervals)
☒	☐	For null hypothesis testing, the test statistic (e.g. *F*, *t*, *r*) with confidence intervals, effect sizes, degrees of freedom and *P* value noted<br>*Give P values as exact values whenever suitable.*
☒	☐	For Bayesian analysis, information on the choice of priors and Markov chain Monte Carlo settings
☒	☐	For hierarchical and complex designs, identification of the appropriate level for tests and full reporting of outcomes
☒	☐	Estimates of effect sizes (e.g. Cohen's *d*, Pearson's *r*), indicating how they were calculated

Our web collection on statistics for biologists contains articles on many of the points above.

Software and code

Policy information about availability of computer code

Data collection	Imgaes and data stored in portable hard drives and on papers.
Data analysis	Images collected using Olympus DSX1000 attached with DSX10-SXLOB10X lens, Nikon SMZ25 attached with SHR Plan APO 0.5x lens, and Sony ILCE-7RM4A attached with 50 mm lens; continental reconstructions generated using GPlates 2.3.0; Social network analysis conducted in RStudio Version 1.3.959; elemental mapping analyzed using ZEISS GeminiSEM 360 attached with ULTIM MAX detector; zircons spiked with EARTHTIME ET535 mixed 205P-233U-235U tracer; U and Pb purified with AG1X-8 anion-exchange resin; U and Pb isotopic ratios measured on an Isotopox X62multicollector thermal ionization mass specktrometer equipped with a Daly photomultiplier ion-counting system; calculation of dates and propagation of uncertainties performed using the Tripoli version 3.7.1 and ET Redux version 0.5.2.; geological map prepared using Adobe Illustrator 26.0.

For manuscripts utilizing custom algorithms or software that are central to the research but not yet described in published literature, software must be made available to editors and reviewers. We strongly encourage code deposition in a community repository (e.g. GitHub). See the Nature Portfolio guidelines for submitting code & software for further information.

Data

Policy information about availability of data

All manuscripts must include a data availability statement. This statement should provide the following information, where applicable:

- Accession codes, unique identifiers, or web links for publicly available datasets
- A description of any restrictions on data availability
- For clinical datasets or third party data, please ensure that the statement adheres to our policy

Fossil specimens (ESEN 0001 - ESEN 0031) and tuff sample (JWJ23-D-01) described in this paper are deposited in the School of Earth Sciences and Engineering at Nanjing University (ESEN) in China and are available for further research by contacting corresponding authros. All study data are included in the article and supplementary information.

Research involving human participants, their data, or biological material

Policy information about studies with human participants or human data. See also policy information about sex, gender (identity/presentation), and sexual orientation and race, ethnicity and racism.

Reporting on sex and gender	*Use the terms sex (biological attribute) and gender (shaped by social and cultural circumstances) carefully in order to avoid confusing both terms. Indicate if findings apply to only one sex or gender; describe whether sex and gender were considered in study design; whether sex and/or gender was determined based on self-reporting or assigned and methods used. Provide in the source data disaggregated sex and gender data, where this information has been collected, and if consent has been obtained for sharing of individual-level data; provide overall numbers in this Reporting Summary. Please state if this information has not been collected. Report sex- and gender-based analyses where performed, justify reasons for lack of sex- and gender-based analysis.*
Reporting on race, ethnicity, or other socially relevant groupings	*Please specify the socially constructed or socially relevant categorization variable(s) used in your manuscript and explain why they were used. Please note that such variables should not be used as proxies for other socially constructed/relevant variables (for example, race or ethnicity should not be used as a proxy for socioeconomic status). Provide clear definitions of the relevant terms used, how they were provided (by the participants/respondents, the researchers, or third parties), and the method(s) used to classify people into the different categories (e.g. self-report, census or administrative data, social media data, etc.) Please provide details about how you controlled for confounding variables in your analyses.*
Population characteristics	*Describe the covariate-relevant population characteristics of the human research participants (e.g. age, genotypic information, past and current diagnosis and treatment categories). If you filled out the behavioural & social sciences study design questions and have nothing to add here, write "See above."*
Recruitment	*Describe how participants were recruited. Outline any potential self-selection bias or other biases that may be present and how these are likely to impact results.*
Ethics oversight	*Identify the organization(s) that approved the study protocol.*

Note that full information on the approval of the study protocol must also be provided in the manuscript.

Field-specific reporting

Please select the one below that is the best fit for your research. If you are not sure, read the appropriate sections before making your selection.

☐ Life sciences ☐ Behavioural & social sciences ☒ Ecological, evolutionary & environmental sciences

For a reference copy of the document with all sections, see nature.com/documents/nr-reporting-summary-flat.pdf

Ecological, evolutionary & environmental sciences study design

All studies must disclose on these points even when the disclosure is negative.

Study description	This study reports a new Ediacaran fossil Lagerstatte, the Tongshan Lagersattte, that preserves the Burgess Shale-type (BST) metazoan fronds, which were previously known only with the Ediacara-type preservation. In addition, the organisms in this biota represent an overlooked deep-water biodiversity that is time-equivalent to the shallow-water Nama Assemblage of the Ediacara Biota. BST preservation of elements of the Ediacara Biota expands the Ediacara biota with a new repository of original compositional information of tissues and teguments to address debates related to the early evolution of animal life.
Research sample	ESEN 0001 to ESEN 0031, JWJ23-D-01
Sampling strategy	Collect specimens in the field and try to collect all samples identified with biological properties. Thousands of specimens were collected in the field and are now available for identifications. Because the fossil materials from this new Lagerstatte include both previously known and new taxa, we have identified these organisms as either belonging to a specific genus/species or as unidentified

	new organisms. Shu-zhong Shen, Zhang-shuai Hou, Xiang-dong Wang and Jin-bo Hou collected zircons.
Data collection	Jin-bo Hou identified fossil materials in the lab, and all co-authors agreed on these identifications. Shu-zhong Shen, Zhang-shuai Hou, and J.R. dated zircons. The stratigraphic information was recorded on paper by Jin-bo Hou in pen. Fossil materials and ash samples were collected using hammer and pick-axes by Jin-bo Hou, Shu-zhong Shen, Xiang-dong Wang, and Zhang-shuai Hou.
Timing and spatial scale	These materials were regularly collected between summer 2022 and spring 2025.
Data exclusions	No data were excluded from the analysis.
Reproducibility	Elemental mapping was successful for all experiments, and each was analyzed once. Zircon dating was conducted on 5 best-well preserved individual zircons, and all analyses were successful.
Randomization	Randomization was not applicable in this study.
Blinding	Blinding was not applicable in this study.

Did the study involve field work? ☒ Yes ☐ No

Field work, collection and transport

Field conditions	After extensively exploring the Dengying Formation in and around the Tongshan County, we selected the Wanjia and Jiweijian sections for detailed study.
Location	All materials were collected at the Wanjia (29°41'46" N, 114°31'30" E) and Jiweijian (29°40'54" N, 114°25'39" E) sections in Tonshan County, Hubei Province, China.
Access & import/export	Materials were collected in Hubei Province and transported to Nanjing University in Jiangsu Province, China. There are no restrictions on transportation between the two provinces. Specimens deposited at School of Earth Sciences and Engineering (ESEN) in Nanjing University are available for further study, and requests for materials should be addressed to Jin-bo Hou or Shu-zhong Shen. We also got the permission of landowner for this scientific study.
Disturbance	There is no disturbance.

Reporting for specific materials, systems and methods

We require information from authors about some types of materials, experimental systems and methods used in many studies. Here, indicate whether each material, system or method listed is relevant to your study. If you are not sure if a list item applies to your research, read the appropriate section before selecting a response.

Materials & experimental systems

n/a	Involved in the study	
☒	☐	Antibodies
☒	☐	Eukaryotic cell lines
☐	☒	Palaeontology and archaeology
☒	☐	Animals and other organisms
☐	☐	Clinical data
☒	☐	Dual use research of concern
☒	☐	Plants

Methods

n/a	Involved in the study	
☒	☐	ChIP-seq
☒	☐	Flow cytometry
☒	☐	MRI-based neuroimaging

Palaeontology and Archaeology

Specimen provenance	Specimens were all collected at the Wanjia and Jiweijian sections in Tongshan County, Hubei Province, China. All material was curated as the School of Earth Sciences and Engineering, Nanjign University, China.
Specimen deposition	All fossil specimens (ESEN 0001 - 0031) and tuff sample (JWJ23-D-01) were deposited at School of Earth Sciences and Engineering (ESEN), Nanjing University.
Dating methods	The tuff sample (JWJ23-D-01) from the Jiweijian Section, about 4.8 m above the Shaanxilithes zone, was analyzed by the U-Pb CA-ID-TIMS method, following the detailed procedures described by Ramezani et al. (18). Zircon separates were extracted from the ash sample using standard crushing, magnetic susceptibility, and high-density liquid techniques. Most zircons are prismatic with delicate glass (melt) inclusions parallel to their long axis under binocular microscope, suggesting a volcanic origin.

The selected zircon grains were pretreated by a chemical abrasion procedure modified after Mattinson(19), which involved thermal annealing in a 900°C furnace for 60 hours, followed by partial dissolution (leaching) in 29M HF inside high-pressure Parr® vessels at 210°C for 12 hours in order to mitigate Pb-loss effects, which often lead to anomalously young dates. The partially dissolved grains underwent fluxing alternately with dilute HNO3 and 6M HCl on a hot plate and in an ultrasonic bath, each for 1 hour. After each ultrasonic step, the grains were rinsed with ultra-pure water to remove the leachates. Subsequently, the thoroughly rinsed zircon grains were spiked with the EARTHTIME ET535 mixed 205Pb-233U-235U tracer(20,21) before complete dissolution in 29M HF at 210°C for 48 hours. The zircon solutions were then dried down on a hot plate and redissolved in 6M HCl inside high-pressure vessels at 180°C overnight. The dissolved U and Pb were chemically purified by an HCl-based column chemistry method using AG1X-8 anion-exchange resin. The eluted U and Pb were dried down with 0.05M H3PO4 and loaded with a silica gel emitter solution onto a zone-refined outgassed Re filament for mass spectrometry.

The U and Pb isotopic ratios were measured on an Isotopx® X62 multi-collector thermal ionization mass spectrometer equipped with a Daly photomultiplier ion-counting system at the Massachusetts Institute of Technology Isotope Laboratory. Pb isotopic ratios were measured as monoatomic Pb ions in a peak-hopping mode on the ion-counter, whereas U isotopes were measured as UO2+ in a static mode on three Faraday detectors simultaneously. Measured isotopic ratios were corrected for mass-dependent isotope fractionation in the mass spectrometer, as well as for U and Pb contributions from the spike and laboratory blanks. Common Pb in the analyses averaged 0.29 pg, all of which was attributed to laboratory blank, and its isotopic composition was determined from long-term measurements of the total procedural Pb blank in the lab (see Supplementary data 1 footnotes). The radiogenic 206Pb concentrations were also corrected for initial 230Th disequilibrium in zircon using a magma Th/U model ratio of 2.8 ± 1.0 (2σ).
Data reduction, calculation of dates and propagation of uncertainties were carried out using the Tripoli and ET_Redux software packages(22,23). Uncertainties in the calculated weighted mean 206Pb/238U date are reported at 2σ level (Extended Data Fig. 2) and in the ± X/Y/Z Ma format, where X is the internal (analytical) uncertainty in the absence of all external errors, Y incorporates X and the U–Pb tracer calibration errors, and Z includes the latter as well as the decay constant errors of(24). The external uncertainties must be taken into account only if the results are compared with U–Pb dates obtained in other laboratories with different tracers, different techniques (e.g. microbeam U–Pb), or ones derived from other isotopic chronometers (e.g. 40Ar/39Ar).

All 5 analyzed zircons from the tuff sample JWJ23-D-01 overlap within their 2σ analytical uncertainties, yielding a weighted mean 206Pb/238U date of 543.74 ± 0.87/0.98/1.1 Ma with a MSWD of 1.0. This date provides the best age estimate for the eruption of the tuff and a good approximation for the associated sedimentary depositional age.

The measurement was conducted at Massachusetts Institute of Technology, USA.

☒ Tick this box to confirm that the raw and calibrated dates are available in the paper or in Supplementary Information.

Ethics oversight: No ethic approval or guidance was required because fossil materials in our study are not involved with this issue.

Note that full information on the approval of the study protocol must also be provided in the manuscript.

Clinical data

Policy information about clinical studies
All manuscripts should comply with the ICMJE guidelines for publication of clinical research and a completed CONSORT checklist must be included with all submissions.

Clinical trial registration: *Provide the trial registration number from ClinicalTrials.gov or an equivalent agency.*

Study protocol: *Note where the full trial protocol can be accessed OR if not available, explain why.*

Data collection: *Describe the settings and locales of data collection, noting the time periods of recruitment and data collection.*

Outcomes: *Describe how you pre-defined primary and secondary outcome measures and how you assessed these measures.*

Plants

Seed stocks: *Report on the source of all seed stocks or other plant material used. If applicable, state the seed stock centre and catalogue number. If plant specimens were collected from the field, describe the collection location, date and sampling procedures.*

Novel plant genotypes: *Describe the methods by which all novel plant genotypes were produced. This includes those generated by transgenic approaches, gene editing, chemical/radiation-based mutagenesis and hybridization. For transgenic lines, describe the transformation method, the number of independent lines analyzed and the generation upon which experiments were performed. For gene-edited lines, describe the editor used, the endogenous sequence targeted for editing, the targeting guide RNA sequence (if applicable) and how the editor was applied.*

Authentication: *Describe any authentication procedures for each seed stock used or novel genotype generated. Describe any experiments used to assess the effect of a mutation and, where applicable, how potential secondary effects (e.g. second site T-DNA insertions, mosiacism, off-target gene editing) were examined.*
